# Transformative experiences at art museums to support flourishing in medicine

**DOI:** 10.1080/10872981.2023.2202914

**Published:** 2023-04-19

**Authors:** Sean Tackett, Lauren Eller, Samuel Scharff, Kamna S. Balhara, Kaitlin M. Stouffer, Melissa Suchanek, Sarah L. Clever, Philip Yenawine, Suzy Wolffe, Margaret S. Chisolm

**Affiliations:** a Division of General Internal Medicine, Johns Hopkins Bayview Medical Center and Biostatistics, Epidemiology, and Data Management Core, Johns Hopkins University School of Medicine, Baltimore, MD, USA; b Johns Hopkins University Bloomberg School of Public Health, Baltimore, MD, USA; c Psychiatry & Behavioral Sciences, Johns Hopkins University School of Medicine, Baltimore, MD, USA; d Emergency Medicine, Johns Hopkins University School of Medicine, Baltimore, MD, USA; e Johns Hopkins University School of Medicine, Baltimore, MD, USA; f Clinical Research Associate, Department of Oncology, Albert Einstein College of Medicine, Bronx, NY, USA; g Department of Medicine and Assistant Dean for Student Affairs, Johns Hopkins University School of Medicine, Baltimore, MD, USA; h Watershed Collaborative, Baltimore, MD, USA; i manager of tour experience, Baltimore, MD, USA; j Psychiatry and Behavioral Sciences, and Medicine, Johns Hopkins University School of Medicine, Baltimore, MD, USA

**Keywords:** Professional identity formation, arts and humanities, visual art, flourishing, wellness

## Abstract

**Purpose:**

We implemented and evaluated a hybrid 4-week arts-based elective for clinical medical students to support flourishing.

**Materials and Methods:**

Five students participated in early 2022. Twelve sessions occurred in-person at art museums and other cultural centers, and five occurred online. Sessions incorporated varied arts-based learning activities, including Visual Thinking Strategies, a jazz seminar, and a mask-making workshop. We evaluated the course via weekly reflective essays, interviews 6 weeks after the course, and pre-post surveys that included four scales with clinical relevance: capacity for wonder (CfW), tolerance for ambiguity (TFA), interpersonal reactivity index, and openness to diversity.

**Results:**

Qualitatively, the course helped learners: 1) reconnect with individual characteristics and interests that had been neglected during medical education; 2) better appreciate others’ perspectives; 3) develop identities as physicians; and 4) engage in quiet reflection, renewing their sense of purpose. Quantitatively, pre-post mean totals increased for the CfW (32.0 [SD 6.8] vs 44.0 [SD 5.7], p=.006) and TFA scales (16.4 [SD 5.2] vs 24.2 [SD 6.9], p=.033).

**Conclusions:**

This elective facilitated learners’ connecting with themselves, others, and their profession with improvement in clinically-relevant measures. This provides further evidence that arts-based education can foster professional identity formation and be transformative for students.

## Introduction

Health is a ‘state of complete physical, mental and social well-being and not merely the absence of disease or infirmity’ [1]. To achieve and sustain true health, physicians need to understand themselves and each of their patients as human beings living within particular social contexts. For both physicians and their patients, the ‘big questions’ (i.e., what it means to be human and to lead a good life) – long considered in Western philosophy – have continued relevance. Aristotle used the term ‘eudamonia,’ best translated as flourishing, to describe the state when all aspects of life are good. The Human Flourishing Program at Harvard’s Institute for Quantitative Social Science, led by Tyler VanderWeele, has reinvigorated the argument for the importance of considering human flourishing in medicine [2]. Developed through analyses of multiple large epidemiologic datasets, VanderWeele’s model of human flourishing includes the well-established components of happiness and life satisfaction, mental and physical health, meaning and purpose, character and virtue, close social relationships, and financial and material stability [3]. Each of these domains satisfies two criteria: it is nearly universally desired and each is an end in itself. To reach these, he has proposed four pathways – family, work, education, and community [4] – which map onto the personal, relational, and professional components of professional identity formation [5].

The alignment between modern concepts of health, professional identity formation, and the notion of flourishing may justify incorporating flourishing into medical curricula. Evidence also suggests that flourishing is linked to well-being in medical learners [6,7]. Supporting student flourishing may be especially important at a time when well-being is likely to be waning during clinical years. This time also serves as a major threshold in the formation of a physician and most US medical schools offer curricula to support this transition [8]. However, the majority of the content in these courses focuses on development of technical knowledge and skills, such as working with the electronic health record, performing clinical procedures, and managing common diseases [9]. Human flourishing tends not to be addressed at all in most of these courses [8].

Medical educators seeking to address learning in the complex domains of flourishing more fully may need to look beyond traditional medical education pedagogies. Like professional identity formation, the teaching of human flourishing may require strategies that allow students to process emotions, explore different perspectives, and question assumptions [10]. When integrated into medical curricula, the arts and humanities can provide opportunities for personal insight, perspective taking, and social advocacy [11,12]. The integration of the arts and humanities into medical curricula has the potential to be transformative [13] and offers a novel way to support the flourishing of clinical-year students on the threshold of change from medical school to residency [14–16].

In this paper, we describe and report results from a 4-week art museum-based course designed to support the flourishing of 3^rd^ and 4^th^ year medical students.

## Materials and methods

### Needs assessment and preliminary work

In the 2019–2020 academic year, we conducted and evaluated six pilot sessions for what we anticipated would be a 4-week in-person art museum-based elective intended to support flourishing in 3^rd^ and 4^th^ year medical students that would take place in February 2021. Pilot sessions revealed both an interest and need for this type of course from learners [17]. After the COVID-19 pandemic closed art museums and shut down in-person teaching, we developed a 1-week online version of the course, which we delivered six times between April 2020 and October 2021 to a total of 42 students [7,18]. Student evaluations of this course indicated that a hybrid of online and in-person was preferred by most.

### Design of the 4-week hybrid elective

We redesigned the 4-week in-person course to include 12 in-person and five online sessions (Table 1). Each week of the course had a theme aligned with curricular objectives (Appendix 1). The first three weeks’ themes – family, community, and work/education – represented the four pathways to human flourishing [4]. The fourth week’s theme – self-care – maps onto the mental and physical health domain of flourishing [4] Each session’s content was unique and included two to three distinct arts-based activities related to these themes (Appendix 2).

**Table 1.** Weekly schedule of elective, February – March 2022.

	Monday	Tuesday	Wednesday	Thursday	Friday
Week 1 Family	Online	Evergreen Museum and Library	Baltimore Museum of Art	Mural Tour of Baltimore City	Online
Week 2 Community	Independent Assignments	Evergreen Museum and Library	Baltimore Museum of Art	Homewood Museum	Online
Week 3 Education and Work	Independent Assignments	Evergreen Museum and Library	Baltimore Museum of Art	Johns Hopkins Hospital	Online
Week 4 Self-Care	Independent Assignments	Cylburn Arboretum	American Visionary Art Museum	Johns Hopkins Hospital & Keystone Korner Jazz Club	Online

### Implementation of the 4-week elective

We delivered the 4-week course February 6 through 4 March 2022 to a group of five 3^rd^ (*n* = 2) and 4^th^ year (*n* = 3) Johns Hopkins University medical students. At this time of the academic calendar, 4^th^ year students would be finalizing their residency match list, and 3^rd^ year students were in the midst of their core clerkships. COVID-19-related museum capacity restrictions limited enrollment to five students. All 3^rd^ and 4^th^ year students were made aware of the course by emails sent from the course director (MC). The course was also visible as part of the online elective course catalogue. Participants were advised that, by enrolling in the course, they were also agreeing to participate in a research study to understand how art museum-based teaching facilitates medical learning. The Johns Hopkins Medicine Institutional Review Board deemed our study protocol exempt (IRB 00210522).

We began each session with a ‘check-in’ to briefly discuss how participants were feeling on that given day and closed each session with a guided written reflection and live reading of a poem or the playing of a recorded song. The two to three art activities varied from session to session, but most sessions included a Visual Thinking Strategies open-ended discussion of facilitator-selected works of visual art or poems [13,19–21], and many sessions included a Personal Responses Tour, in which learners selected a work of art from a gallery in response to either shared or unique prompts [13,22,23]. (M. Kelly-Hedrick et al. [24] Other experiences included:

Back-to-back sketching – a ‘describer’ faces a work of art unseen by the ‘drawer’ who attempts to recreate the object based on detailed verbal descriptions from their partner [13];

Group poems – teams of learners iteratively create a written work based on shared visual and spoken prompts [13];

Forest bathing – individuals slowly move through a natural landscape, cultivating a sense of presence by noticing what they see and hear [25];

Mask-making – individuals create masks to express elements of self in the context of their experiences in medical education (led by guest facilitator Mark Stephens) [26,27];

Jazz seminar – learners participate in a series of listening and viewing exercises aimed at using jazz to develop one’s own authentic ‘voice’ (led by guest facilitator Paul Haidet) [28]).

We graded the course as pass/fail, with 70% of the grade based on students’ overall participation. At the start of the course, we stated that we expected all students to participate in most activities. Of the 5 students, 2 had perfect attendance, 1 missed 1 online session, 1 missed 1 in-person session, and 1 missed 2 in-person sessions (all missed in-person sessions were due to an individual’s COVID-19 exposure or illness precluding their in-person course attendance).

### Data collection

Reflective writing. All students completed five 750-word minimum writing assignments. After the first session in week 1, we posted the prompt for assignment #1 on the course Blackboard site asking about expectations for their experiences in the course (Appendix 4); assignment #1 was due on Blackboard prior to the second session, which occurred the following day. We posted the prompts for assignments #2–5 on Blackboard after the final session of each week; each assignment was due prior to the start of the following week. Each weekly prompt asked them to reflect on how the course had influenced them that week (Appendix 4). Once uploaded to Blackboard, the course director de-identified the writing assignments, replacing each student’s name on the assignment with their unique participant ID number, prior to storing the de-identified assignments on the secure research site. The course director then read, commented on, and graded each student assignment on Blackboard as pass/fail based on receipt by deadline and achievement of minimum word count within 2 days of receipt.

Follow-up interviews. Approximately 6 weeks after the course ended and all final grades for the course had been received by the registrar’s office, the course director emailed each student inviting them to email a study team member (LE) who was not part of the teaching team if they were interested in opting into a 30-minute structured interview about their experience in the course. All 5 students opted in to participation. Each interview was conducted via the Zoom videoconferencing platform. In the interview, LE asked each student several questions (Appendix 5) about their experiences in the course, topics addressed by the course, and how the course compared to their medical school curriculum as a whole. LE used no student names or other identifiers during the interview. Audio recordings of interviews were transcribed by automated transcription software.

Pre-post surveys. Prior to the first session of the course, the course director sent each student an email containing a unique participant ID number that they used to complete a pre-course Qualtrics survey whose link was sent via a separate email. (Appendix 3). The pre-course survey included six items on the participant’s background in the arts and humanities, as well as four scales – each containing seven to 10 items with Likert-type response options. We selected these scales based on: (1) the clinical relevance of the attributes they measured [29–33], (2) previous studies suggesting the impact of arts and humanities learning activities on these attributes [14,15], and (3) availability of published means and validity evidence for these scales from college student [31] or large U.S. medical student samples [29,30,32,33].

The four scales are (1) Capacity for Wonder (CfW) scale (10 items, with two subscales – perspective shifting and emotional reawakening) [31,32]; (2) Tolerance for Ambiguity (TFA) scale (7 items) [29,33]; (3) Interpersonal Reactivity Index (IRI) measure of empathy (8 items, limited to perspective taking and empathic concern subdomains of IRI) [30,33]; and (4) Openness to diversity scale (9 items) [33].

The course director sent a link at the end of the last session to the post-course survey, which included all four scales and six additional items: four asking for ratings of course quality and relevance and two open-ended prompts seeking comments on positive aspects of the course and suggestions for improvement (Appendix 3).

### Data analysis

We aggregated each participant’s written reflections and two study team members (ST, LE) completed a preliminary thematic analysis. During this preliminary analysis, LE started conducting the follow-up interviews, with the written reflection thematic analysis iteratively informing the interviews. LE then performed a preliminary reading of the interview transcripts, in which she identified no themes distinct from those in the written reflections. Thus, we decided to analyze the written reflections and interviews as one dataset. We used the qualitative software package NVivo (release 1.5.1, 2021) to facilitate data management and organization. We first developed codes inductively to categorize the data, then combined these codes to identify larger themes; analytic memos aided in the process of collapsing codes into larger themes. Two study team members (ST, LE) met several times during the process to discuss and refine the analytic approach, reviewed the final themes and quotes, and achieved consensus on their final presentation.

We performed descriptive statistics on pre- and post-course survey items. We compared differences for individual’s scale totals post- vs pre-course survey ratings using paired t-tests.

## Results

Of the five medical students enrolled in this elective, most had actively engaged with literary arts (*n* = 4) prior to the course. Fewer engaged with music (*n* = 2), visual arts (*n* = 2), film (*n* = 1), or dance (*n* = 1). Two had taken a museum-based course previously, and one majored and one minored in an arts and humanities field as an undergraduate.

Our qualitative analysis of the written reflections and interviews identified four overarching themes. We list the themes, their definitions, and illustrative quotes in Table 3. Briefly, participants described how the course’s learning activities allowed them to: 1) reconnect with their individual characteristics and interests, which they felt had been neglected or dormant during their medical education; 2) connect with and better appreciate the perspectives of one another; 3) develop their identities as physicians; and 4) engage in quiet reflection on art and their medical education experiences, which renewed their sense of purpose.

Results of pre- and post-course responses for the four scales are shown in Table 2. We found statistically significant increases for mean totals for the CfW (32.0 [SD 6.8] vs 44.0 [SD 5.7], *p* = .006) and TFA scales (16.4 [SD 5.2] vs 24.2 [SD 6.9], *p* = .033). Each individual showed increases pre- to post-course for CfW and TFA scales. Changes for IRI and openness to diversity scales did not reach statistical significance (*p* > .05).

**Table 2.** Comparisons of 5 individuals’ responses for scales included on pre and post course evaluation surveys.

	Pre	Post	
	Mean (SD)	Mean (SD)	P value
Capacity for wonder (CfW)	32.0 (6.8)	44.0 (5.7)	0.006
Tolerance for ambiguity (TFA)	16.4 (5.2)	24.2 (6.9)	0.033
Interpersonal Reactivity Index (IRI)	21.6 (3.6)	23.2 (2.3)	0.347
Openness to diversity scale	21.6 (6.8)	25.0 (7.0)	0.179

CfW range is 5–50; TFA range is 0–35; IRI range is 0–32; Openness to diversity scale range is 0–45.

In post-course surveys, all five participants endorsed being ‘extremely likely’ to recommend the course to a friend and ‘strongly agreed’ that the course was relevant to their clinical work, personal life, and work beyond the clinical encounter (e.g., in research, administration, policy development, and/or community service).

## Discussion

Third- and fourth-year medical students enrolled in this 4-week hybrid online and in-person art museum-based elective valued the course as a way to reconnect with self and others, mature in their professional identity formation, and find a renewed sense of purpose. We also found measurable improvements in clinically relevant constructs by the end of the course. Our results provide further support that the integration of the arts and humanities into medical education can be transformative for students.

The arts and humanities have a fundamental role to play in medical education and offer many opportunities for innovation in teaching and learning [14,15]. Although most publications describing or evaluating the arts and humanities in medical education have focused on skill mastery in pre-clinical medical students, these programs can also support personal insight, perspective taking, and social advocacy at all stages of education and training [11,12,16]. We designed this art museum-based course to support the flourishing of medical students by selecting a time in their development when they have sufficient exposure to the culture of medicine to have an experiential basis for reflection and are ready to look ahead to the physician they seek to become. We further supported learning by aligning with evidence-based practices – providing creative outlets, reflective opportunities, and a social environment that fostered honesty and intimacy [10]. Students indicated in reflective writing and interviews that this design helped them discover new aspects of themselves and grow personally and professionally. The variety of contexts, from art museums to outdoor walking reflection, may also have helped facilitate perspective transformation.

Given the limited statistical power with our small sample, only dramatic changes in our pre-post survey measures would register as statistically significant. Yet, we found significant increases in participants’ wonder capacity and ambiguity tolerance measures. CfW is important as it has been associated with empathy in medical students [33] and may support the development of virtues such as humility and courage [32]. TFA is a clinically relevant attribute: lower TFA has been associated with physician avoidance of certain kinds of patients (those with lower socioeconomic status or a history of substance use, for example), a higher need for cognitive closure, fear of making mistakes, increased test ordering behavior, discomfort with death/grief, and other physician attitudes and practices [33].

We do not know precisely what was responsible for the nearly uniformly positive influence of the course’s experiences on students, but we are aware that the course design was distinct from reported arts and humanities-based courses in a number of ways. First, the class size, necessarily limited due to COVID-19 restrictions, was relatively small compared to other arts and humanities programs and may have allowed for the building of greater intimacy and trust [14,15]. Second, the 4-week duration was relatively long compared to other such programs [14,15]. Third, the use of art museums and natural settings for the majority of the synchronous sessions was unusual for most programs [14,15]; their quiet separateness and relative unfamiliarity may have created the ‘sacred space’ and ‘disorienting dilemma’ needed for transformation [13,34]. Fourth, online sessions allowed the course designers to select from a greater variety and number of art objects; the selection of specific objects may have been influential. Fifth, the incorporation of creating activities may have greater impact than more passive beholding activities. Finally, access to specially trained and experienced medical and museum educators [14,15] may have elicited stronger responses from participants.

The mask making workshop is worth highlighting because every participants’ reflective essays provided striking descriptions of their impact (only a handful of which we were able to include as illustrative quotes in Table 3). This workshop was held in an art museum classroom (very similar to a medical school classroom, where it can also be held) and it represented the longest creative activity held during the course (a full hour was spent creating one’s mask). Although our workshop featured a medical educator from another institution with expertise in this activity and the field of museum-based education, the supplies for this learning activity are easily accessible and the methods have been well-described [26]. Of all of our activities included in our elective, mask-making may be the most feasible for educators without special training while also offering a significant opportunity for students to reflect on their identities and purpose.

**Table 3.** Themes, definitions, and illustrative quotes from reflective writing and interviews of course participants.

Theme	Definition of theme	Example quotes
Art to connect with oneself	Creative activities and dialogue helped participants discover new things about themselves and reconnect with parts of themselves that had been dormant	“ … designing the mural, drawing the couple at the hospital, mask making and creating a piece to represent thresholds … allowed me to engage some of the artistic skills I had developed prior to medical school, that have gone a bit more out of practice in the last few years.” “I remembered the joy in life that I felt and re-experienced those emotions during the writing session. It reminded me that there was joy and valuable experiences in life no matter what my outcome on an exam turns out to be.” “As I went through this process, I was surprised by how much I saw myself in the art work, and how much I got a chance to learn more about myself through it, as if each of these artworks represented a tiny piece of mirror reflecting different parts of myself, and that I was trying to capture of all these scattered reflections to make sense of who I am as a person.”
Art to connect with others and their perspectives	Analyzing art individually and as a class helped participants appreciate other perspectives and increase empathy	“I believe this appreciation for hearing other’s perspectives and incorporating their thoughts into my own interpretations is one of the major ways this course impacted me.” “There have … been times in which a peers’ observation is novel to me, encouraging me to look at something from a different angle. I hope to continue to practice this mental flexibility … , while possibly applying various forms of perspective taking as I face what feels like a large life/career decision.” “The activity really helped me form different perspectives on people’s inner worlds, how they can be constructed, and how we can strive to understand them through different means.”
Art to foster professional identity formation	Participants contemplated their existing and future identities as physicians and the process of identity formation by analogy to creative activities	“Dissonance is distracting, and wearing a mask draws from our limited sources of energy … . Going forward, I am grateful to have an additional way of thinking about this: ‘What does my external mask show? What does my internal mask show? What are their similarities, and why are there differences?’.” “[Jazz performers] communication while they played … [had me compare] jazz performers process through which they find their particular musical voice to physicians finding their own communication style and voice. It helped me reflect on what my voice as a physician will sound like and how I should develop that voice.” “Throughout medical school so far, I think the emphasis has been on trying to transform the inner self into something that is closer to the fixed, ideal outer self. However, from our discussion … I gained a different perspective that the transformation can go both ways simultaneously.”
Time to be free of distractions and achieve mindfulness	Quiet and reflective course experiences provided opportunities for participants to process experiences and re-examine purpose	“ … the solo museum tours and walk through the Clyburn Arboretum were particularly impactful because they helped me experience and learn techniques that encourage being in the moment and minimizing distractions. Because I was given a specific task for each of these experiences I had a strong sense of purpose while conducting these activities and had to focus on the task at hand rather than think about distractions.” “I certainly had many reflective experiences during clinical clerkships, particularly in grave or especially joyous situations, but I’m not sure I had much time to reflect on my overall purpose of going into, and continuing in, medicine. But in this course, when we were often viewing art depicting very human experiences and asked to draw connections to medicine, we had the space to start some of that deeper reflection.” “Just being able to sit and focus on expressing how I feel about my medical school experience through art was regenerative to me. Having that time to reflect on my medical professional journey helped me realize that I have a lot more hope for myself and my professional endeavors than I realized. When simply talking about how I feel about my professional life without deeply reflecting, I paint a lot of despondency and disillusion into my narrative.”

Important limitations must be considered. First, the sample size was small and the elective nature of the course predisposed this study to self-selection bias. We could not account for baseline characteristics that may have influenced students’ responses or known whether most students would experience this course as positively as those who chose to take it. Our statistical power was limited to detect differences in empathy and openness to diversity measures, and our design does not allow us to explain why certain scale measures changed more than others. Second, several of the sessions were facilitated by guest educators who were well-versed in the exercise they were facilitating. Such expert facilitators may not be available to all programs. However, there has been significant growth in faculty development opportunities designed to enhance educator comfort with arts- and humanities-based pedagogies [8,35,36]. Third, and similarly, not all settings may have access to the full breadth of museums and cultural spaces that we did; it is likely, however, that most settings do have some analogous resources, which could include local art galleries or street art. Additionally, online sessions can be easily adapted to any setting. Finally, the course combined a variety of activities and we are not able to determine if one element was more important than another or if additive effects were experienced.

This unique hybrid online and in-person 4-week art museum-based elective for 3^rd^ and 4^th^ year medical students showed promise for improving clinically-relevant measures and helping students connect with themselves and others and progress in their professional development. Grounded in an evidence-based model of human flourishing, it has the potential to enhance the well-being and flourishing of physicians and thus improve the health of the patients they serve.

## Supplementary Material

Supplemental Material

## Appendix 1. Course Learning Objectives

1. Facilitate deepened student reflection on what it means to be human, to be a physician, and to lead a good life (for oneself and one’s patients).
2. Facilitate student reflection on one’s sense of self in relation to one’s family, community, work, and education experiences.
3. Facilitate student reflection on how family, community, education, and work experiences offer opportunities for improving one’s life satisfaction and happiness, physical and mental health, character and virtue, meaning and purpose, and close social relationships.
4. Facilitate student reflection on the role of the arts and humanities in mastering skills, appreciating multiple perspectives, gaining personal insight, and supporting social advocacy.
5. Facilitate student reflection on how the arts and humanities can support self-care and wellbeing.

## Appendix 2. Week-by-Week Daily Session Schedule

**Table:**

Week 1 (Family)	Session 1	Session 2	Session 3	Session 4	Session 5
Location	Online	Evergreen Museum and Library	Baltimore Museum of Art	Baltimore City Communities	Online
Art Activity 1	Personal Responses Tour with Introductions Prompt: Choose an image from the slide deck that says something meaningful about yourself and discuss it with your partner.	Visual Thinking Strategies of Painting *Interior*/Vuillard	Close Looking at Sculpture *Three Piece Reclining Figure No. 1*/Moore	Visual Thinking Strategies of Murals *Love in Search of a Word*/Bergner *African Prince*/Ozmo	Visual Thinking Strategies of Poem *In the smallest corner of worlds*/Spicer
Art Activity 2	Course Overview	Discuss Homework	Personal Responses Tour with Gift Creation in Sculptures Garden Prompt: Select a figurative sculpture and craft from art materials a gift that might help you in your daily life	Visual Thinking Strategies of Murals *Four Women with Different Relationships to Property*/Gaia *One Day at a Time/*Owen	Discuss Homework
Art Activity 3	Visual Thinking Strategies of Painting *Christina’s World/*Wyeth	Personal Responses Tour of Mr. Garrett’s Library Prompt: Find an object that offers a glimpse of your inner world	Discuss Homework	Discuss Homework	Creation of a Group Poem *Emma 1*/Stoehr Group A engages in the assignment with the artist’s text included in the image Group B engages in the activity in response to image with artist’s text redacted
Written Reflection	Describe what it feels like to say your name	Write about a home you’ve left behind	Write about a shoulder you lean on	Describe a seed of yourself you’ve planted	Describe where you wear your heart
Closing Poem/Song	*For a New Beginning*/O’Donohue	*The Lanyard*/Collins	*Lament*/St. Vincent Millay	*Color*/Harrington	*Father and Son*/Yusuf
HW Assignment	Writing Assignment #1 and read *Gateways to Wonder*/O’Donohue	Find or create an object that offers a perspective on your relationship to your family	Create a black and white sketch of a mural you’d create to represent your family	Create a color version of the mural sketch you created yesterday	Writing Assignment #2 and reflect on the quote: ‘We perceive only what we’ve learned to look for, both in life and in art.’/Albert C Barnes

**Table:**

Week 2 (Community)	Session 6	Session 7	Session 8	Session 9
Location	Evergreen Museum and Library	Baltimore Museum of Art	Homewood Museum	Online
Activity 1	Visual Thinking Strategies of Video Installation *Proscenium/*Brown	Close Looking *Great Mother Headdress (D’mba)*/Unknown	Museum Tour	Visual Thinking Strategies of Photograph *Untitled (Man Smoking/Malcolm X*)/Weems
Activity 2	Discuss Homework	Sketching Activity *Forest*/Mosley Draw the sculpture from any angle, then compile the sketches and compare them. How does working together help you see more than you would alone?	Discuss Homework	Discuss Longitudinal Assignment #1 Independent VTS Session at Johns Hopkins Hospital Prompt: Sketch two people you see together, paying attention to non-verbal cues that suggest the nature of their relationship
Activity 3	Personal Responses Tour of Garrett Boys’ Den Prompt: Find an object that you haven’t seen before that interests or influences you	Discuss Homework	Visual Thinking Strategies of Painting *J Marion Sims: Gynecologic Surgeon*/Thom	Discuss Homework
Written Reflection Prompt	Describe your home that isn’t a house	Describe a stranger you know	Write about a mark you cannot erase	Describe an unspoken conversation you’ve had
Closing Poem/Song	*The Family*/Oliver	*The Eskimos Have No Word for War*/Oliver	*The Researcher Discovers Anarcha, Betsey, Lucy*/Judd	*Mercy Now*/Gauthier
HW Assignment	Create something that offers a glimpse of how you define and relate to your community	Read the article: *How Doctors Take Women’s Pain Less Seriously*/Fassler	Watch the trailer for *Pina/*Wenders Select a scene and reflect on the relationship between at least two of the dancers	Writing Assignment #3 and watch *Rivers and Tides: Andy Goldsworthy Working with Time/*Riedelsheimer

**Table:**

Week 3 (work/education)	Session 10	Session 11	Session 12	Session 13
Location	Evergreen Museum and Library	Baltimore Museum of Art	Johns Hopkins Hospital	Online
Activity 1	Personal Responses Tour of Parlor/Foyer Galleries Prompt: Select a work that uses an artistic style in which you yourself would like to be portrayed	Back-to-Back Sketching Activity Divide into pairs. Student A looks at *Paysage (A Winter Day in Brittany)*/Picknell and describes it to their partner, Student B Student B looks away from the painting and draws what they hear Repeat with *A Wild Scene* Cole, switching roles How does careful word choice affect what others hear?	Discuss Homework	Visual Thinking Strategies of Painting *Painting for My Father/*Noah Davis
Activity 2	Discuss Homework		Visual Thinking Strategies of Painting *Untitled* /Martin and Muñoz	Video Activity *Streb and the Art of Extreme Action Movement*/THNKR
Activity 3	Mask-Making Activity	Quadrant Activity Select a work that speaks to their purpose in medicine. Now sketch it, reflect on it, and imagine what it would sound like as a song	Scavenger Hunt Activity Students identify artwork throughout the hospital that connects with multiple prompts	Fear Discussion Students reflect on their potential concerns about a career in medicine
Written Reflection	Describe what you recognize in the face in the mirror. What do you not?	Write about your pride in another’s accomplishment	Write about a time you wandered into wonder	Write about a time you walked on uneven ground
Closing Poem/Song	*Love after Love*/Wolcott	*Eagle or Sun Part XIV*/Paz	*The Journey*/Oliver	*I Go Among Trees*/Berry
HW Assignment	n/a	Read the article: *This institution was never meant for me*/Fitzsousa et Al.	Read an excerpt of *On Being interview transcript*/O’Donohue	Writing Assignment #4 and reminder that longitudinal assignments 2 and 3 are due the following week

**Table:**

Week 4 (self-care)	Session 14	Session 15	Session 16	Session 17
Location	Cylburn Arboretum	American Visionary Art Museum	JHH Lecture Hall & Keystone Korner Jazz Club	Online
Activity 1	Discuss Longitudinal Assignment #2 Personal Responses Tour at the Rawlings Conservatory Prompt: Identify two plants, one that you could nourish and the other that could nourish you	Visual Thinking Strategies of Painting *Untitled/*Snodgrass	Jazz and the Art of Medicine – using jazz to develop one’s own authentic ‘voice’	Visual Thinking Strategies of Poem *In Memory of My Feelings – Frank O’Hara/*Johns
Activity 2	Forest Bath and Personal Responses Tour of Forest Walk the Blue Border Trail at Cylburn Arboretum. How does the forest floor feel beneath your feet? What does the air taste like? Next, find a place on the trail to stand still in nature, apart from others. Identify something you can see but cannot hear. Take a photograph or make a sketch of it. From the same place, find things that you can hear but not see. Record a ‘voice memo’ of what you discover.	Personal Responses Tour of Sculptures *Time Markers/*Josephson Select one bust that best represents the way you coped with the pandemic.		Discuss Longitudinal Assignment #3 Personal Responses Tour at the Walters Art Museum At session 2, each student randomly selected a unique prompt for which they were instructed to find a work of art that – for them – responds to the prompt
Activity 3		Creation of a Group Poem *Dylann Roof*/Haughton Group A engages in the activity in response to the image and its accompanying description Group B engages in the activity in response to the image alone	Visual Thinking Strategies of Photograph *Between Takes at Birdland/*le Querec	Post-Course Survey
Written Reflection Prompt	Write about a day in the life of your hands	Write about a time you were healed with a fall	Write about a voice that’s never quiet	Write about a time you were present through your absence
Closing Poem/Song	*Work, Sometimes*/Oliver	*I am not I*/Jiménez (trans. Bly)	*Thought-work*/O’Donohue	*Absence*/O’Donohue
HW Assignment	Read an excerpt from *The Well-Gardened Mind*/Stuart-Smith Engage in an activity that brought you comfort during quarantine	Select a piece of music you find nourishing or comforting during a period of transition	Evening Jazz Concert at Keystone Korner (optional)	Writing Assignment #5

## Appendix 3. Pre-and Post-course Surveys

**Pre-course Survey**

Q0. User ID Number

Q1. Please carefully read each statement and indicate how likely you are to:

**Table:**

	Extremely unlikely	Somewhat unlikely	Neither likely or unlikely	Somewhat likely	Extremely likely
Find yourself drawing new connections between things in the world					
Take to heart experiences that challenge your understanding of the world					
Be described by others as inquisitive					
Find yourself pausing to reflect					
Move among several different perspectives on the same situation like a camera or microscope lens zooming in and out					

Q2. Please carefully read each statement and indicate how likely you are to:

**Table:**

	Extremely unlikely	Somewhat unlikely	Neither likely or unlikely	Somewhat likely	Extremely likely
Experience familiar things as if for the first time					
Feel amazement during the ordinary course of events					
Feel personally engaged by an experience that takes your breath away					
See the world with an interest of a child					
Experience surprise					

Q3:Please indicate the extent to which you agree with the following statements:

**Table:**

	Strongly disagree	Moderately disagree	Slightly disagree	Slightly agree	Moderately agree	Strongly agree
It really disturbs me when I am unable to follow another person’s train of thought.						
If I am uncertain about the responsibilities involved in a particular task, I get very anxious.						
Before any important task, I must know how long it will take.						
I don’t like to work on a problem unless there is a possibility of getting a clear-cut and unambiguous answer.						
The best part of working on a jigsaw puzzle is putting in that last piece.						
I am often uncomfortable with people unless I feel that I can understand their behavior.						
A good task is one in which what is to be done and how it is to be done is always clear.						

Q4. The following statements inquire about your thoughts and feelings in a variety of situations. For each item, indicate how well it describes you by choosing the appropriate number on the scale: 1, 2, 3, 4, or 5. Read each item carefully before responding. Answer as honestly as you can.

**Table:**

	1 (does not describe me well)	2	3	4	5 (describes me very well)
I often have tender, concerned feelings for people less fortunate than me.					
Other people’s misfortunes do not usually disturb me a great deal.					
I am often quite touched by things that I see happen.					
I would describe myself as a pretty soft-hearted person.					
I sometimes try to understand my friends better by imagining how things look from their perspective.					
When I’m upset at someone, I usually try to “put myself in their shoes” for a while.					
I try to look at everybody’s side of a disagreement before I make a decision.					
Before criticizing somebody, I try to imagine how I would feel if I were in their place.					

Q5. During your medical experience, how often did you gain a deeper understanding of other perspectives through conversations with fellow students because:

**Table:**

	Never	Rarely	Occasionally	Somewhat often	Often	Very often
Their religious beliefs were different from yours						
Their political opinions were different from yours						
Their nationality was different from yours						
Their primary language was different from yours						
Their race or ethnicity was different from yours						
Their sexual orientation was different from yours						
Their socioeconomic background was different from yours						
Their physical abilities were different from yours						
Their age was different from yours						

Q6. Which kinds of art, if any, do you actively participate in? (check all that apply)

- Music
- Film
- Literature
- Visual arts
- Dance
- Theater arts
- Other (please specify)

Q7. Have you incorporated music, film, literature, visual arts, dance, and/or theater arts into your work at JHMI – for example, as part of patient care, teaching, research, or community engagement?

- Yes
- No

Q8. Prior to this course, have you engaged in any other arts-based experiences as part of your medical education at Johns Hopkins? (Both formal and informal experiences count.)

- Yes
- No

Q9. Did you major in an arts or humanities discipline as an undergraduate?

- Yes
- No

Q10. Did you minor in an arts or humanities discipline as an undergraduate?

- Yes
- No

Q11. Have you ever taken a museum-based course in the past?

- Yes
- No

**Post-course survey**

Q0. User ID Number

Q1. Please carefully read each statement and indicate how likely you are to:

**Table:**

	Extremely unlikely	Somewhat unlikely	Neither likely or unlikely	Somewhat likely	Extremely likely
Find yourself drawing new connections between things in the world					
Take to heart experiences that challenge your understanding of the world					
Be described by others as inquisitive					
Find yourself pausing to reflect					
Move among several different perspectives on the same situation like a camera or microscope lens zooming in and out					

Q2. Please carefully read each statement and indicate how likely you are to:

**Table:**

	Extremely unlikely	Somewhat unlikely	Neither likely or unlikely	Somewhat likely	Extremely likely
Experience familiar things as if for the first time					
Feel amazement during the ordinary course of events					
Feel personally engaged by an experience that takes your breath away					
See the world with an interest of a child					
Experience surprise					

Q3:Please indicate the extent to which you agree with the following statements:

**Table:**

	Strongly disagree	Moderately disagree	Slightly disagree	Slightly agree	Moderately agree	Strongly agree
It really disturbs me when I am unable to follow another person’s train of thought.						
If I am uncertain about the responsibilities involved in a particular task, I get very anxious.						
Before any important task, I must know how long it will take.						
I don’t like to work on a problem unless there is a possibility of getting a clear-cut and unambiguous answer.						
The best part of working on a jigsaw puzzle is putting in that last piece.						
I am often uncomfortable with people unless I feel that I can understand their behavior.						
A good task is one in which what is to be done and how it is to be done is always clear.						

Q4. The following statements inquire about your thoughts and feelings in a variety of situations. For each item, indicate how well it describes you by choosing the appropriate number on the scale: 1, 2, 3, 4, or 5. Read each item carefully before responding. Answer as honestly as you can.

**Table:**

	1 (does not describe me well)	2	3	4	5 (describes me very well)
I often have tender, concerned feelings for people less fortunate than me.					
Other people’s misfortunes do not usually disturb me a great deal.					
I am often quite touched by things that I see happen.					
I would describe myself as a pretty soft-hearted person.					
I sometimes try to understand my friends better by imagining how things look from their perspective.					
When I’m upset at someone, I usually try to ‘put myself in their shoes’ for a while.					
I try to look at everybody’s side of a disagreement before I make a decision.					
Before criticizing somebody, I try to imagine how I would feel if I were in their place.					

Q5. During your medical experience, how often did you gain a deeper understanding of other perspectives through conversations with fellow students because:

**Table:**

	Never	Rarely	Occasionally	Somewhat often	Often	Very often
Their religious beliefs were different from yours						
Their political opinions were different from yours						
Their nationality was different from yours						
Their primary language was different from yours						
Their race or ethnicity was different from yours						
Their sexual orientation was different from yours						
Their socioeconomic background was different from yours						
Their physical abilities were different from yours						
Their age was different from yours						

Q6. How likely are you to recommend this course to a friend?

- Not at all likely
- Somewhat likely
- Extremely likely

Q7. Please indicate the extent to which you agree that the skills you developed in this course may be relevant to various aspects of your personal and professional development:

**Table:**

	Strongly disagree	Slightly disagree	Neutral	Slightly agree	Strongly agree
Relevant to clinical work?					
Relevant to personal life?					
Relevant to work beyond the clinical encounter – in research, administration, policy development, and/or community service?					

Q8. What aspects of this course went particularly well?[Free response]Q9. How can the course improve for next time?[Free response]

## Appendix 4. Written Assignments

Assignment 1 (after first day of course)

What are you looking forward to as you begin this course? How do you think participating in this course might influence you?

Assignments 2-5 (after each week of course)

Reflecting on the previous week’s course activities, select one or more activity that influenced you. What about this/them makes you say it/they influenced you? Is there anything about your experience in the course so far that has surprised you?

Assignment 6 (after last day of course)

Looking back over the whole course, how did you change? What activities had the greatest influence (for better or worse) and what about them makes you say they influenced you? What activities didn’t have a strong influence on you, and what about them makes you say they didn’t influence you?

## Appendix 5. Follow-up Interview Questions

How did this art museum-based course compare to your previous medical school curricular experiences?

Did this course address important topics that had not already been addressed in your curriculum?

If so, what makes you think those topics were important, and how did the course address them?

Were some topics that were addressed in this course already part of your formal medical school curriculum? How did the way these topics were addressed in this course compare?

If they don’t mention the following topics:

I noticed you did not mention the topics of [empathy, wonder, tolerance for ambiguity, diversity] being a gap in your medical school curriculum. Is that because you feel this topic was sufficiently addressed in your other courses? If not, do you think this course added in any way to your understanding of this topic?

Wrap-up question:

Is there anything more that you’d like to say about how this course addressed any topics in your medical school curriculum?
