# Supplementary material for: Transformative experiences at art museums to support flourishing in medicine
Source: Med Educ Online. 2023 Apr 19;28(1):2202914. doi: 10.1080/10872981.2023.2202914 (PMC10120547; doi:10.1080/10872981.2023.2202914)
Supplement: Supplemental Material [file ZMEO_A_2202914_SM6929.docx]

Appendices

Appendix 1. Course Learning Objectives

1. Facilitate deepened student reflection on what it means to be human, to be a physician, and to lead a good life (for oneself and one’s patients).
2. Facilitate student reflection on one’s sense of self in relation to one’s family, community, work, and education experiences.
3. Facilitate student reflection on how family, community, education, and work experiences offer opportunities for improving one’s life satisfaction and happiness, physical and mental health, character and virtue, meaning and purpose, and close social relationships.
4. Facilitate student reflection on the role of the arts and humanities in mastering skills, appreciating multiple perspectives, gaining personal insight, and supporting social advocacy.
5. Facilitate student reflection on how the arts and humanities can support self-care and wellbeing.

Appendix 2. Week-by-Week Daily Session Schedule

| **Week 1**  **(Family)** | **Session 1** | **Session 2** | **Session 3** | **Session 4** | **Session 5** |
| --- | --- | --- | --- | --- | --- |
| **Location** | **Online** | **Evergreen Museum and Library** | **Baltimore Museum of Art** | **Baltimore City Communities** | **Online** |
| **Art Activity 1** | **Personal Responses Tour with Introductions**  Prompt: Choose an image from the slide deck that says something meaningful about yourself and discuss it with your partner. | **Visual Thinking Strategies of Painting**  *Interior*/Vuillard | **Close Looking at Sculpture**  *Three Piece Reclining Figure No. 1*/ Moore | **Visual Thinking Strategies of Murals**  *Love in Search of a Word*/Bergner  *African Prince*/Ozmo | **Visual Thinking Strategies of Poem**  *In the smallest corner of worlds*/Spicer |
| **Art Activity 2** | **Course Overview** | **Discuss Homework** | **Personal Responses Tour with Gift Creation in Sculptures Garden**  Prompt: Select a figurative sculpture and craft from art materials a gift that might help you in your daily life | **Visual Thinking Strategies of Murals**  *Four Women with Different Relationships to Property*/Gaia  *One Day at a Time/*Owen | **Discuss Homework** |
| **Art Activity 3** | **Visual Thinking Strategies of Painting**  *Christina’s World/*Wyeth | **Personal Responses Tour of Mr. Garrett’s Library**  Prompt: Find an object that offers a glimpse of your inner world | **Discuss Homework** | **Discuss Homework** | **Creation of a Group Poem**  *Emma 1*/ Stoehr  Group A engages in the assignment with the artist’s text included in the image  Group B engages in the activity in response to image with artist’s text redacted |
| **Written Reflection** | Describe what it feels like to say your name | Write about a home you’ve left behind | Write about a shoulder you lean on | Describe a seed of yourself you’ve planted | Describe where you wear your heart |
| **Closing Poem/Song** | *For a New Beginning*/ O’Donohue | *The Lanyard*/Collins | *Lament*/St. Vincent Millay | *Color*/Harrington | *Father and Son*/Yusuf |
| **HW Assignment** | Writing Assignment #1 and read *Gateways to Wonder*/O’Donohue | Find or create an object that offers a perspective on your relationship to your family | Create a black and white sketch of a mural you’d create to represent your family | Create a color version of the mural sketch you created yesterday | Writing Assignment #2 and reflect on the quote: “We perceive only what we’ve learned to look for, both in life and in art.”/Albert C Barnes |

| **Week 2**  **(Community)** | **Session 6** | **Session 7** | **Session 8** | **Session 9** |
| --- | --- | --- | --- | --- |
| **Location** | **Evergreen Museum and Library** | **Baltimore Museum of Art** | **Homewood**  **Museum** | **Online** |
| **Activity 1** | **Visual Thinking Strategies of Video Installation**  *Proscenium/*Brown | **Close Looking**  *Great Mother Headdress (D'mba)*/Unknown | **Museum Tour** | **Visual Thinking Strategies of Photograph**  *Untitled (Man Smoking/Malcolm X*)/ Weems |
| **Activity 2** | **Discuss Homework** | **Sketching Activity**  *Forest*/Mosley  Draw the sculpture from any angle, then compile the sketches and compare them. How does working together help you see more than you would alone? | **Discuss Homework** | **Discuss Longitudinal Assignment #1**  Independent VTS Session at Johns Hopkins Hospital  Prompt: Sketch two people you see together, paying attention to non-verbal cues that suggest the nature of their relationship |
| **Activity 3** | **Personal Responses Tour of Garrett Boys’ Den**  Prompt: Find an object that you haven’t seen before that interests or influences you | **Discuss Homework** | **Visual Thinking Strategies of Painting**  *J Marion Sims: Gynecologic Surgeon*/Thom | **Discuss Homework** |
| **Written Reflection Prompt** | Describe your home that isn’t a house | Describe a stranger you know | Write about a mark you cannot erase | Describe an unspoken conversation you’ve had |
| **Closing Poem/Song** | *The Family*/Oliver | *The Eskimos Have No Word for War*/Oliver | *The Researcher Discovers Anarcha, Betsey, Lucy*/Judd | *Mercy Now*/ Gauthier |
| **HW Assignment** | Create something that offers a glimpse of how you define and relate to your community | Read the article: *How Doctors Take Women’s Pain Less Seriously*/Fassler | Watch the trailer for *Pina/* Wenders  Select a scene and reflect on the relationship between at least two of the dancers | Writing Assignment #3 and watch *Rivers and Tides: Andy Goldsworthy Working with Time/*Riedelsheimer |

| **Week 3**  **(work/education)** | **Session 10** | **Session 11** | **Session 12** | **Session 13** |
| --- | --- | --- | --- | --- |
| **Location** | **Evergreen Museum and Library** | **Baltimore Museum of Art** | **Johns Hopkins Hospital** | **Online** |
| **Activity 1** | **Personal Responses Tour of Parlor/Foyer Galleries**  Prompt: Select a work that uses an artistic style in which you yourself would like to be portrayed | **Back-to-Back Sketching Activity**  Divide into pairs. Student A looks at *Paysage (A Winter Day in Brittany)* / Picknell and describes it to their partner, Student B  Student B looks away from the painting and draws what they hear  Repeat with *A Wild Scene* Cole, switching roles  How does careful word choice affect what others hear? | **Discuss Homework** | **Visual Thinking Strategies of Painting**  *Painting for My Father/*Noah Davis |
| **Activity 2** | **Discuss Homework** |  | **Visual Thinking Strategies of Painting**  *Untitled* / Martin and Muñoz | **Video Activity**  *Streb and the Art of Extreme Action Movement*/ THNKR |
| **Activity 3** | **Mask-Making Activity** | **Quadrant Activity**  Select a work that speaks to their purpose in medicine. Now sketch it, reflect on it, and imagine what it would sound like as a song | **Scavenger Hunt Activity**  Students identify artwork throughout the hospital that connects with multiple prompts | **Fear Discussion**  Students reflect on their potential concerns about a career in medicine |
| **Written Reflection** | Describe what you recognize in the face in the mirror. What do you not? | Write about your pride in another’s accomplishment | Write about a time you wandered into wonder | Write about a time you walked on uneven ground |
| **Closing Poem/Song** | *Love after Love*/Wolcott | *Eagle or Sun Part XIV*/Paz | *The Journey*/Oliver | *I Go Among Trees*/Berry |
| **HW Assignment** | n/a | Read the article: *This institution was never meant for me*/Fitzsousa et Al. | Read an excerpt of *On Being interview transcript*/O’Donohue | Writing Assignment #4 and reminder that longitudinal assignments 2 and 3 are due the following week |

| **Week 4**  **(self-care)** | **Session 14** | **Session 15** | **Session 16** | **Session 17** |
| --- | --- | --- | --- | --- |
| **Location** | **Cylburn Arboretum** | **American Visionary Art Museum** | **JHH Lecture Hall & Keystone Korner Jazz Club** | **Online** |
| **Activity 1** | **Discuss Longitudinal Assignment #2**  Personal Responses Tour at the Rawlings Conservatory  Prompt: Identify two plants, one that you could nourish and the other that could nourish you | **Visual Thinking Strategies of Painting**  *Untitled/*Snodgrass | **Jazz and the Art of Medicine - using jazz to develop one’s own authentic “voice”** | **Visual Thinking Strategies of Poem**  *In Memory of My Feelings – Frank O’Hara/*Johns |
| **Activity 2** | **Forest Bath and Personal Responses Tour of Forest**  Walk the Blue Border Trail at Cylburn Arboretum. How does the forest floor feel beneath your feet? What does the air taste like?  Next, find a place on the trail to stand still in nature, apart from others. Identify something you can see but cannot hear. Take a photograph or make a sketch of it. From the same place, find things that you can hear but not see. Record a ‘voice memo’ of what you discover. | **Personal Responses Tour of Sculptures**  *Time Markers/*Josephson  Select one bust that best represents the way you coped with the pandemic. |  | **Discuss Longitudinal Assignment #3**  Personal Responses Tour at the Walters Art Museum  At session 2, each student randomly selected a unique prompt for which they were instructed to find a work of art that – for them - responds to the prompt |
| **Activity 3** |  | **Creation of a Group Poem**  *Dylann Roof*/Haughton  Group A engages in the activity in response to the image and its accompanying description  Group B engages in the activity in response to the image alone | **Visual Thinking Strategies of Photograph**  *Between Takes at Birdland/*le Querec | **Post-Course Survey** |
| **Written Reflection Prompt** | Write about a day in the life of your hands | Write about a time you were healed with a fall | Write about a voice that’s never quiet | Write about a time you were present through your absence |
| **Closing Poem/Song** | *Work, Sometimes*/Oliver | *I am not I*/ Jiménez (trans. Bly) | *Thought-work*/ O’Donohue | *Absence*/O’Donohue |
| **HW Assignment** | Read an excerpt from *The Well-Gardened Mind*/Stuart-Smith  Engage in an activity that brought you comfort during quarantine | Select a piece of music you find nourishing or comforting during a period of transition | Evening Jazz Concert at Keystone Korner (optional) | Writing Assignment #5 |

Appendix 3

Pre-and Post-course Surveys

**Pre-course Survey**

Q0. User ID Number

Q1. Please carefully read each statement and indicate how likely you are to:

|  | Extremely unlikely | Somewhat unlikely | Neither likely or unlikely | Somewhat likely | Extremely likely |
| --- | --- | --- | --- | --- | --- |
| Find yourself drawing new connections between things in the world |  |  |  |  |  |
| Take to heart experiences that challenge your understanding of the world |  |  |  |  |  |
| Be described by others as inquisitive |  |  |  |  |  |
| Find yourself pausing to reflect |  |  |  |  |  |
| Move among several different perspectives on the same situation like a camera or microscope lens zooming in and out |  |  |  |  |  |

Q2. Please carefully read each statement and indicate how likely you are to:

|  | Extremely unlikely | Somewhat unlikely | Neither likely or unlikely | Somewhat likely | Extremely likely |
| --- | --- | --- | --- | --- | --- |
| Experience familiar things as if for the first time |  |  |  |  |  |
| Feel amazement during the ordinary course of events |  |  |  |  |  |
| Feel personally engaged by an experience that takes your breath away |  |  |  |  |  |
| See the world with an interest of a child |  |  |  |  |  |
| Experience surprise |  |  |  |  |  |

Q3: Please indicate the extent to which you agree with the following statements:

|  | Strongly disagree | Moderately disagree | Slightly disagree | Slightly agree | Moderately agree | Strongly agree |
| --- | --- | --- | --- | --- | --- | --- |
| It really disturbs me when I am unable to follow another person’s train of thought. |  |  |  |  |  |  |
| If I am uncertain about the responsibilities involved in a particular task, I get very anxious. |  |  |  |  |  |  |
| Before any important task, I must know how long it will take. |  |  |  |  |  |  |
| I don’t like to work on a problem unless there is a possibility of getting a clear-cut and unambiguous answer. |  |  |  |  |  |  |
| The best part of working on a jigsaw puzzle is putting in that last piece. |  |  |  |  |  |  |
| I am often uncomfortable with people unless I feel that I can understand their behavior. |  |  |  |  |  |  |
| A good task is one in which what is to be done and how it is to be done is always clear. |  |  |  |  |  |  |

Q4. The following statements inquire about your thoughts and feelings in a variety of situations. For each item, indicate how well it describes you by choosing the appropriate number on the scale: 1, 2, 3, 4, or 5. Read each item carefully before responding. Answer as honestly as you can.

|  | 1  (does not describe me well) | 2 | 3 | 4 | 5 (describes me very well) |
| --- | --- | --- | --- | --- | --- |
| I often have tender, concerned feelings for people less fortunate than me. |  |  |  |  |  |
| Other people’s misfortunes do not usually disturb me a great deal. |  |  |  |  |  |
| I am often quite touched by things that I see happen. |  |  |  |  |  |
| I would describe myself as a pretty soft-hearted person. |  |  |  |  |  |
| I sometimes try to understand my friends better by imagining how things look from their perspective. |  |  |  |  |  |
| When I’m upset at someone, I usually try to “put myself in their shoes” for a while. |  |  |  |  |  |
| I try to look at everybody’s side of a disagreement before I make a decision. |  |  |  |  |  |
| Before criticizing somebody, I try to imagine how I would feel if I were in their place. |  |  |  |  |  |

Q5. During your medical experience, how often did you gain a deeper understanding of other perspectives through conversations with fellow students because:

|  | Never | Rarely | Occasionally | Somewhat often | Often | Very often |
| --- | --- | --- | --- | --- | --- | --- |
| Their religious beliefs were different from yours |  |  |  |  |  |  |
| Their political opinions were different from yours |  |  |  |  |  |  |
| Their nationality was different from yours |  |  |  |  |  |  |
| Their primary language was different from yours |  |  |  |  |  |  |
| Their race or ethnicity was different from yours |  |  |  |  |  |  |
| Their sexual orientation was different from yours |  |  |  |  |  |  |
| Their socioeconomic background was different from yours |  |  |  |  |  |  |
| Their physical abilities were different from yours |  |  |  |  |  |  |
| Their age was different from yours |  |  |  |  |  |  |

Q6. Which kinds of art, if any, do you actively participate in? (check all that apply)

- Music
- Film
- Literature
- Visual arts
- Dance
- Theater arts
- Other (please specify)

Q7. Have you incorporated music, film, literature, visual arts, dance, and/or theater arts into your work at JHMI – for example, as part of patient care, teaching, research, or community engagement?

- Yes
- No

Q8. Prior to this course, have you engaged in any other arts-based experiences as part of your medical education at Johns Hopkins? (Both formal and informal experiences count.)

- Yes
- No

Q9. Did you major in an arts or humanities discipline as an undergraduate?

- Yes
- No

Q10. Did you minor in an arts or humanities discipline as an undergraduate?

- Yes
- No

Q11. Have you ever taken a museum-based course in the past?

- Yes
- No

**Post-course survey**

Q0. User ID Number

Q1. Please carefully read each statement and indicate how likely you are to:

|  | Extremely unlikely | Somewhat unlikely | Neither likely or unlikely | Somewhat likely | Extremely likely |
| --- | --- | --- | --- | --- | --- |
| Find yourself drawing new connections between things in the world |  |  |  |  |  |
| Take to heart experiences that challenge your understanding of the world |  |  |  |  |  |
| Be described by others as inquisitive |  |  |  |  |  |
| Find yourself pausing to reflect |  |  |  |  |  |
| Move among several different perspectives on the same situation like a camera or microscope lens zooming in and out |  |  |  |  |  |

Q2. Please carefully read each statement and indicate how likely you are to:

|  | Extremely unlikely | Somewhat unlikely | Neither likely or unlikely | Somewhat likely | Extremely likely |
| --- | --- | --- | --- | --- | --- |
| Experience familiar things as if for the first time |  |  |  |  |  |
| Feel amazement during the ordinary course of events |  |  |  |  |  |
| Feel personally engaged by an experience that takes your breath away |  |  |  |  |  |
| See the world with an interest of a child |  |  |  |  |  |
| Experience surprise |  |  |  |  |  |

Q3: Please indicate the extent to which you agree with the following statements:

|  | Strongly disagree | Moderately disagree | Slightly disagree | Slightly agree | Moderately agree | Strongly agree |
| --- | --- | --- | --- | --- | --- | --- |
| It really disturbs me when I am unable to follow another person’s train of thought. |  |  |  |  |  |  |
| If I am uncertain about the responsibilities involved in a particular task, I get very anxious. |  |  |  |  |  |  |
| Before any important task, I must know how long it will take. |  |  |  |  |  |  |
| I don’t like to work on a problem unless there is a possibility of getting a clear-cut and unambiguous answer. |  |  |  |  |  |  |
| The best part of working on a jigsaw puzzle is putting in that last piece. |  |  |  |  |  |  |
| I am often uncomfortable with people unless I feel that I can understand their behavior. |  |  |  |  |  |  |
| A good task is one in which what is to be done and how it is to be done is always clear. |  |  |  |  |  |  |

Q4. The following statements inquire about your thoughts and feelings in a variety of situations. For each item, indicate how well it describes you by choosing the appropriate number on the scale: 1, 2, 3, 4, or 5. Read each item carefully before responding. Answer as honestly as you can.

|  | 1  (does not describe me well) | 2 | 3 | 4 | 5 (describes me very well) |
| --- | --- | --- | --- | --- | --- |
| I often have tender, concerned feelings for people less fortunate than me. |  |  |  |  |  |
| Other people’s misfortunes do not usually disturb me a great deal. |  |  |  |  |  |
| I am often quite touched by things that I see happen. |  |  |  |  |  |
| I would describe myself as a pretty soft-hearted person. |  |  |  |  |  |
| I sometimes try to understand my friends better by imagining how things look from their perspective. |  |  |  |  |  |
| When I’m upset at someone, I usually try to “put myself in their shoes” for a while. |  |  |  |  |  |
| I try to look at everybody’s side of a disagreement before I make a decision. |  |  |  |  |  |
| Before criticizing somebody, I try to imagine how I would feel if I were in their place. |  |  |  |  |  |

Q5. During your medical experience, how often did you gain a deeper understanding of other perspectives through conversations with fellow students because:

|  | Never | Rarely | Occasionally | Somewhat often | Often | Very often |
| --- | --- | --- | --- | --- | --- | --- |
| Their religious beliefs were different from yours |  |  |  |  |  |  |
| Their political opinions were different from yours |  |  |  |  |  |  |
| Their nationality was different from yours |  |  |  |  |  |  |
| Their primary language was different from yours |  |  |  |  |  |  |
| Their race or ethnicity was different from yours |  |  |  |  |  |  |
| Their sexual orientation was different from yours |  |  |  |  |  |  |
| Their socioeconomic background was different from yours |  |  |  |  |  |  |
| Their physical abilities were different from yours |  |  |  |  |  |  |
| Their age was different from yours |  |  |  |  |  |  |

Q6. How likely are you to recommend this course to a friend?

- Not at all likely
- Somewhat likely
- Extremely likely

Q7. Please indicate the extent to which you agree that the skills you developed in this course may be relevant to various aspects of your personal and professional development:

|  | Strongly disagree | Slightly disagree | Neutral | Slightly agree | Strongly agree |
| --- | --- | --- | --- | --- | --- |
| Relevant to clinical work? |  |  |  |  |  |
| Relevant to personal life? |  |  |  |  |  |
| Relevant to work beyond the clinical encounter – in research, administration, policy development, and/or community service? |  |  |  |  |  |

Q8. What aspects of this course went particularly well?

[Free response]

Q9. How can the course improve for next time?

[Free response]

**Appendix 4. Written Assignments**

**Assignment 1 (after first day of course)**

What are you looking forward to as you begin this course? How do you think participating in this course might influence you?

**Assignments 2-5 (after each week of course)**

**Reflecting on the previous week’s course activities, select one or more activity that influenced you.** **What about this/them makes you say it/they influenced you?** **Is there anything about your experience in the course so far that has surprised you?**

Assignment 6 (after last day of course)

**Looking back over the whole course, how did you change?** **What activities had the greatest influence (for better or worse) and what about them makes you say they influenced you?** **What activities didn’t have a strong influence on you, and what about them makes you say they didn’t influence you?**

Appendix 5. Follow-up Interview Questions

How did this art museum-based course compare to your previous medical school curricular experiences?

Did this course address important topics that had not already been addressed in your curriculum?

If so, what makes you think those topics were important, and how did the course address them?

Were some topics that were addressed in this course already part of your formal medical school curriculum? How did the way these topics were addressed in this course compare?

If they don’t mention the following topics:

I noticed you did not mention the topics of [empathy, wonder, tolerance for ambiguity, diversity] being a gap in your medical school curriculum. Is that because you feel this topic was sufficiently addressed in your other courses? If not, do you think this course added in any way to your understanding of this topic?

Wrap-up question:

Is there anything more that you’d like to say about how this course addressed any topics in your medical school curriculum?
